# HPV-18 E2 protein downregulates antisense noncoding mitochondrial RNA-2, delaying replicative senescence of human keratinocytes

**DOI:** 10.18632/aging.101711

**Published:** 2018-12-30

**Authors:** Claudio Villota, Manuel Varas-Godoy, Emanuel Jeldes, América Campos, Jaime Villegas, Vincenzo Borgna, Luis O. Burzio, Verónica A. Burzio

**Affiliations:** 1 Fundación Ciencia & Vida, Santiago, Chile; 2 Andes Biotechnologies SpA, Santiago, Chile; 3 Departamento de Ciencias Químicas y Biológicas, Facultad de Salud, Universidad Bernardo O’Higgins, Santiago, Chile; 4 Centro de Investigación Biomédica, Facultad de Medicina, Universidad de los Andes, Santiago, Chile; 5 Departamento de Ciencias Biológicas, Facultad de Ciencias de la Vida, Universidad Andrés Bello, Santiago, Chile; 6 Laboratorio de Comunicaciones Celulares (CEMC) Facultad de Medicina, Universidad de Chile, Santiago, Chile; 7 Faculty of Medical Sciences, Universidad de Santiago de Chile, Santiago, Chile; * Equal contribution

**Keywords:** senescence, G2 arrest, mitochondrial ncRNAs, cervical cancer, HPV-18 E2

## Abstract

Human and mouse cells display a differential expression pattern of a family of mitochondrial noncoding RNAs (ncmtRNAs), according to proliferative status. Normal proliferating and cancer cells express a sense ncmtRNA (SncmtRNA), which seems to be required for cell proliferation, and two antisense transcripts referred to as ASncmtRNA-1 and -2. Remarkably however, the ASncmtRNAs are downregulated in human and mouse cancer cells, including HeLa and SiHa cells, transformed with HPV-18 and HPV-16, respectively. HPV E2 protein is considered a tumor suppressor in the context of high-risk HPV-induced transformation and therefore, to explore the mechanisms involved in the downregulation of ASncmtRNAs during tumorigenesis, we studied human foreskin keratinocytes (HFK) transduced with lentiviral-encoded HPV-18 E2. Transduced cells displayed a significantly extended replicative lifespan of up to 23 population doublings, compared to 8 in control cells, together with downregulation of the ASncmtRNAs. At 26 population doublings, cells transduced with E2 were arrested at G_2_/M, together with downregulation of E2 and SncmtRNA and upregulation of ASncmtRNA-2. Our results suggest a role for high-risk HPV E2 protein in cellular immortalization. Additionally, we propose a new cellular phenotype according to the expression of the SncmtRNA and the ASncmtRNAs.

## Introduction

The Human Papillomavirus (HPV) is the etiological agent of cervical cancer, the second most common cancer in women worldwide [1,2]. About 60-70% of cervical cancers are associated to high-risk HPV-16 and HPV-18 [1,2]. The HPV genome encodes six early proteins (E1, E2, E4, E5, E6 and E7), with regulatory functions, and two late proteins (L1 and L2) with structural functions [2–4]. HPV E6 and E7 oncoproteins induce malignant transformation of HPV-infected cells, while HPV E1 and E2 proteins are involved in viral replication, along with the establishment of long-term infection [2–4]. HPV E2 has been described as a negative regulator of E6/E7 transcription and usually the E2 open reading frame disruption is considered a prior stage to cell transformation [5–7]. Currently, E2 is considered a tumor suppressor in the context of HPV-induced transformation [8–10].

Human and mouse cells express a unique family of sense and antisense mitochondrial ncRNAs [11–17]. The sense transcript (SncmtRNA) is expressed in normal proliferating cells and tumor cells but not in resting cells, suggesting a correlation between SncmtRNA expression and cell cycle progression [11,12,14,15]. Indeed, silencing of SncmtRNA with antisense oligonucleotides in HeLa and SiHa cells (transformed with HPV-18 and HPV-16, respectively) induces inhibition of cell proliferation [14,15]. Moreover, stimulation of naïve lymphocytes with phytohaemagglutinin (PHA) induces DNA synthesis, together with expression of PCNA and SncmtRNA. When PHA stimulation is carried out together with rhodamine 6G, a drug that disables mitochondrial function and inhibits cell proliferation [18], DNA synthesis and expression of proliferating cell nuclear antigen (PCNA) and SncmtRNA are inhibited, which is reversed upon removal of the drug [12]. Normal proliferating cells also express two antisense transcripts, ASncmtRNA-1 and -2 [12]. In contrast, in tumor cell lines, as well as tumor cells in different human cancer biopsies, expression of the ASncmtRNAs is downregulated [12–17]. These observations suggest that downregulation of the ASncmtRNAs is an essential step during neoplastic transformation and progression, representing an additional hallmark of cancer [12,19]. Transfection of antisense oligonucleotides of several human and mouse tumor cell lines, independent of the tissue origin, induces massive apoptotic death [15–17]. Moreover, *in vivo* syngeneic studies with B16F10 murine melanoma and RenCa murine renal carcinoma cells showed that the ASncmtRNAs are potent targets to inhibit tumor growth and metastasis [16,17].

However, one pending question is which cellular factor(s) is(are) involved in downregulation of the expression of ASncmtRNAs during oncogenic transformation. As an approach to address this question, here we studied normal human foreskin keratinocytes (HFK) transduced with a lentiviral construct encoding HPV-18 E2. As described before, E2 protein is considered a tumor suppressor [8–10] and therefore it was reasonable to hypothesize that this viral protein could be involved in downregulation of ASncmtRNAs during high-risk HPV-induced oncogenic transformation. Transduced cells showed a significant extension of replicative lifespan, from 8 to 23 population doublings, while ASncmtRNAs were concomitantly downregulated. At population doubling or passage 26 (p26), and together with downregulation of E2, the cells became senescent and arrested at G_2_/M, while ASncmtRNA-2 was upregulated. On the other hand, SncmtRNA was downregulated, supporting the notion that this transcript plays a regulatory function in cell proliferation.

## RESULTS

### HFK-E2 cells express the HPV-18 E2 oncoprotein and downregulate ASncmtRNAs

**Figure 1:**
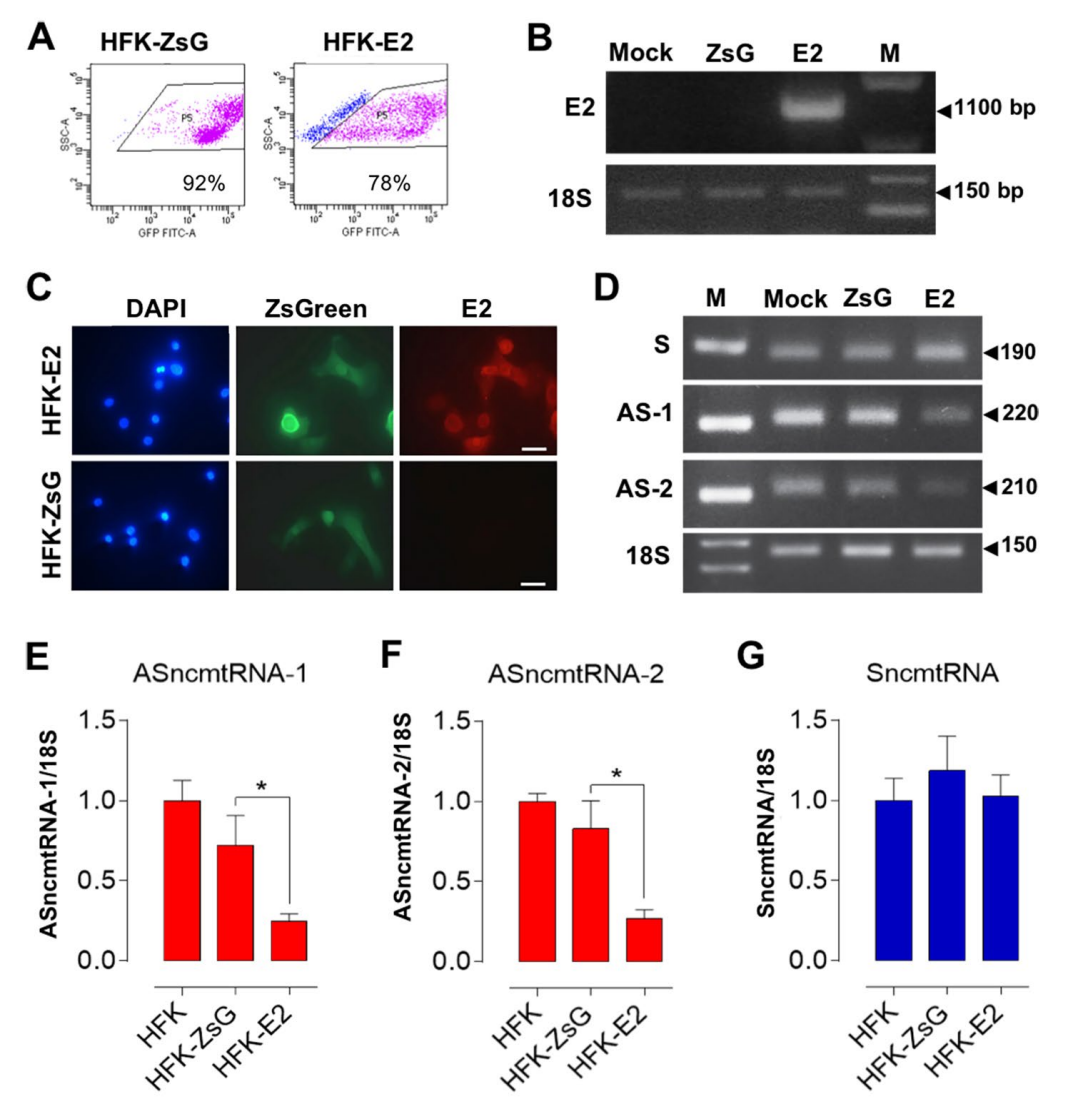
**E2-expressing HFK downregulate the ASncmtRNAs.** HFK were transduced in triplicate for 72 h with HPV-18 E2 (HFK-E2) or with control lentivirus (HFK-ZsG) or mock-transduced. (**A**) Representative analysis of HFK-ZsG and HFK-E2 populations displayed 92% and 78% transduction, respectively. (**B**) Only HFK-E2 cells expressed the full-length E2 mRNA (1,100 bp). (**C**) Only HPV-18 E2-transduced HFK expressed E2 protein as evaluated by immunofluorescence (Bars = 40 µm). (**D**) Relative levels of ncmtRNAs were determined at p3 by RT-PCR using 18S rRNA as loading control. Numbers on the right denote amplicon size in bp. Triplicate analysis of ASncmtRNA-1 (**E**), ASncmtRNA-2 (**F**) and SncmtRNA (**G**) showed that both ASncmtRNAs were downregulated by E2 expression (*p<0.01), while SncmtRNA levels remained unchanged.

To establish whether the E2 protein alone induces downregulation of the ASncmtRNAs in an HPV-negative context, we transduced HFK with lentiviral constructs encoding the green fluorescent protein ZsGreen alone (HFK-ZsG) or ZsGreen and HPV-18 E2 (HFK-E2) and purified cells by sorting, obtaining on the average transduced cell populations of 92% and 78%, respectively (Fig. 1A). Only those cells transduced with HFK-E2 expressed E2 mRNA (Fig. 1B). Immunofluorescence confirmed that only HFK-E2 cells express the E2 protein, localized to the cytoplasm (Fig.1C). Moreover, at population doubling or passage 3 (p3), only HFK-E2 cells showed downregulation of both ASncmtRNAs (Fig. 1D, E, F), while the expression of SncmtRNA was unaffected (Fig. 1D, G).

### E2 oncoprotein extends replicative lifespan of HFK

**Figure 2:**
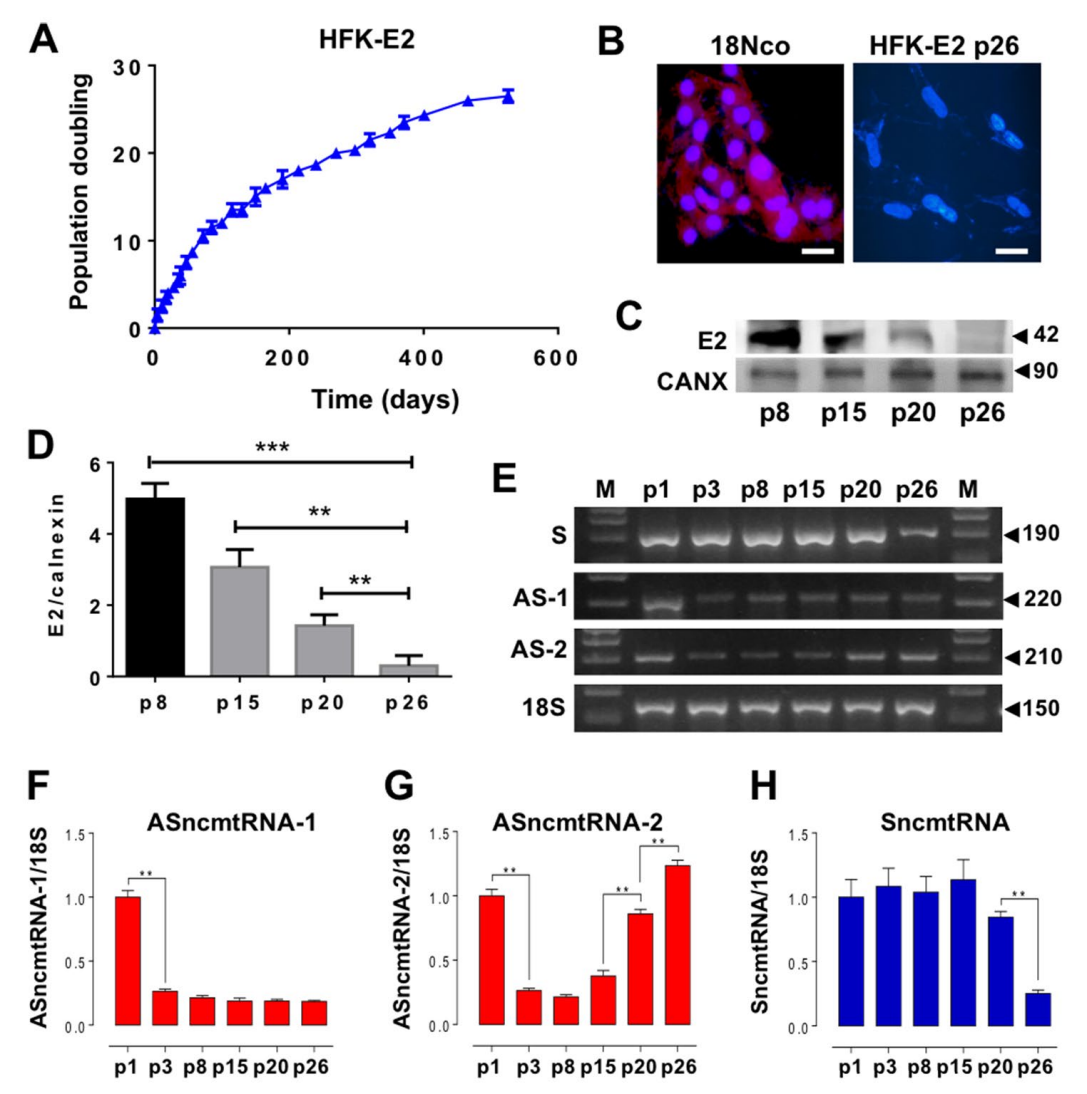
**HPV-18 E2 expression extends replicative lifespan of HFK and modulates expression of ncmtRNAs.** (**A**) A triplicate determination of proliferation rate was plotted as time (days) versus cell doubling or passage number (p). Cells were arrested at p26. (**B**) At p26 the expression of HPV-18 E2 was suppressed as compared to 18Nco cells, transduced with the complete genome of HPV-18 (Bars = 50 µm). (**C**) E2 protein expression was progressively reduced in HFK-E2, from p8 to p26. Calnexin (CANX) was used as loading control. (**D**) A triplicate analysis of the results in (C) show a quantification of the gradual decrease of E2 from p8 to p26 (**p=0.001; ***p=0.0001). (**E**) Determination of the relative expression of ncmtRNAs by RT-PCR using 18S rRNA as loading control. A triplicate analysis shows downregulation of ASncmtRNA-1 at p3 (**p<0.01), which is sustained until p26 (**F**). In contrast, ASncmtRNA-2 is also downregulated at p3 but is later progressively upregulated from p15 to senescence at p26 (**p=0.001) (**G**). (**H**) SncmtRNA is downregulated at p26 (**p=0.001).

To evaluate whether HPV-18 E2 is able to induce HFK immortalization, population doubling of transduced cells was measured at different passages. A triplicate determination showed that HFK-E2 cells maintained proliferative activity until becoming arrested at p26 (Fig. 2A). At this stage, expression of E2 was downregulated, as determined by fluorescent immunocytochemistry, compared to HFK transduced with the complete genome of HPV-18 (18Nco cells), used as positive control for E2 expression [14] (Fig 2B). Western blot carried out in triplicate confirmed the progressive downregulation of E2 (Fig. 2C, D). At p3 after E2 transduction, cells strongly downregulated ASncmtRNA-1 and this low expression level remained constant until replicative arrest at p26 (Fig. 2E, F). In contrast, ASncmtRNA-2 also decreased at p3 but gradually increased from p15 up until p26 arrest, where its expression was recovered to the levels found in early passage HFK-E2 cells (Fig. 2E, G). Interestingly also, at p26 HFK-E2 cells downregulated the expression of SncmtRNA (Fig. 2E, H), supporting the hypothesis that this transcript is involved in cell cycle progression [11,12,14,15].

### E2-expressing HFK arrest at G_2_/M and become senescent

**Figure 3:**
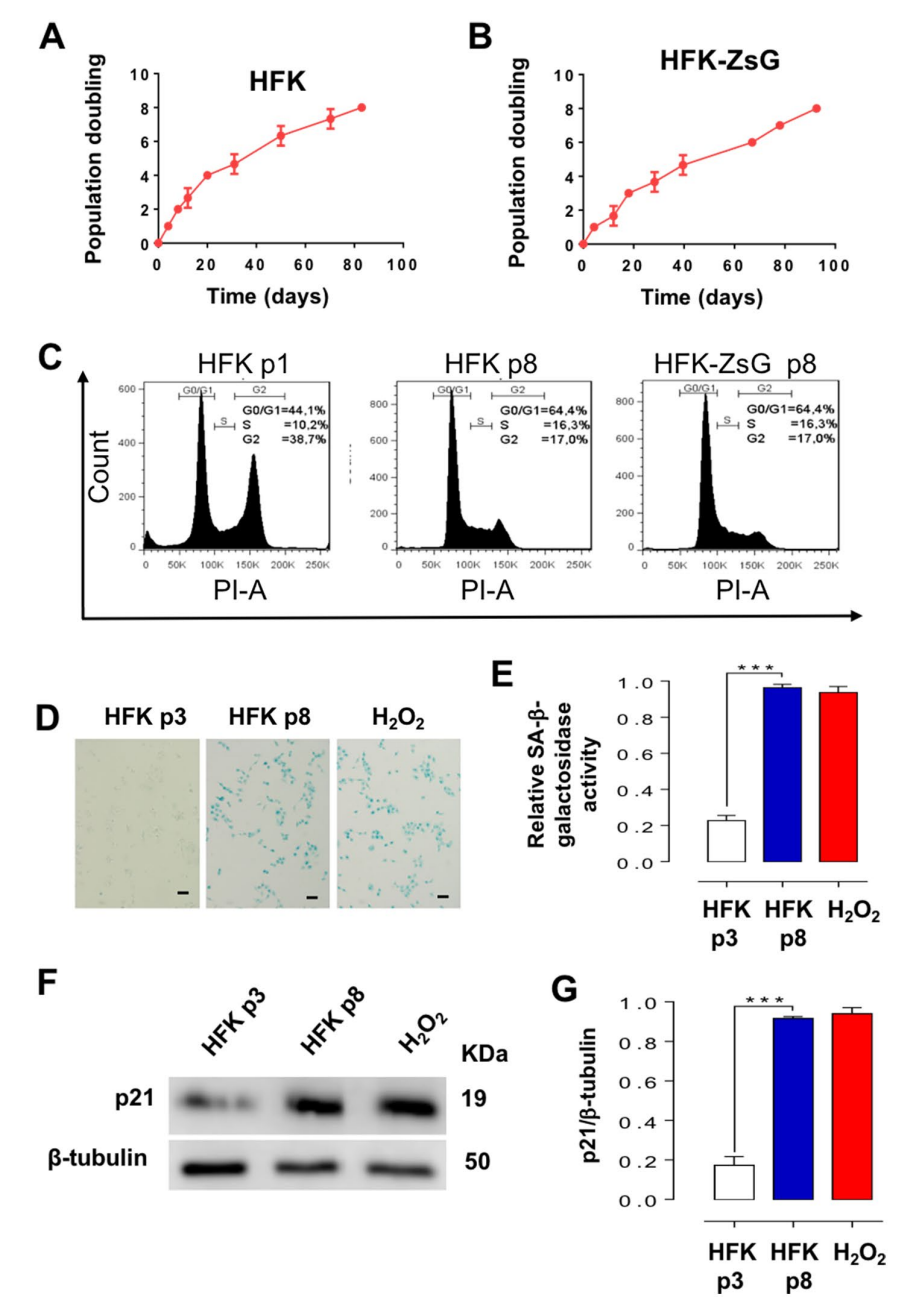
**HFK and HFK-ZsG undergo senescent arrest in G_1_ at p8.** (**A, B**) A triplicate analysis of proliferation rate of HFK and HFK-ZsG was plotted as time (days) versus cell doubling or passage number. Neither cell population proliferated past p8. (**C**) Flow cytometry of HFK at p1 and p8 and HFK-ZsG at p8 shows that non-E2-expressing cells arrest in G_1_ at p8. (**D**) HFK at p8 were positive for SA-β-galactosidase staining, which was negative in p3 cells. H_2_O_2_ was used as positive senescence control (Bars = 100 μm). (**E**) A triplicate analysis of the results shown in D revealed a 4 to 5-fold increase in SA-β-galactosidase activity, compared to p3 cells, and similar to the level of HFK treated with H_2_O_2_ (***p<0.005). (**F**) In HFK at p8, p21 was upregulated compared to p3 cells. (**G**) A triplicate analysis of the experiment in F revealed a 4 to 5-fold increase in p21 levels compared to p3 cells, and at a similar level to H_2_O_2_-treated cells (***p<0.005).

**Figure 4:**
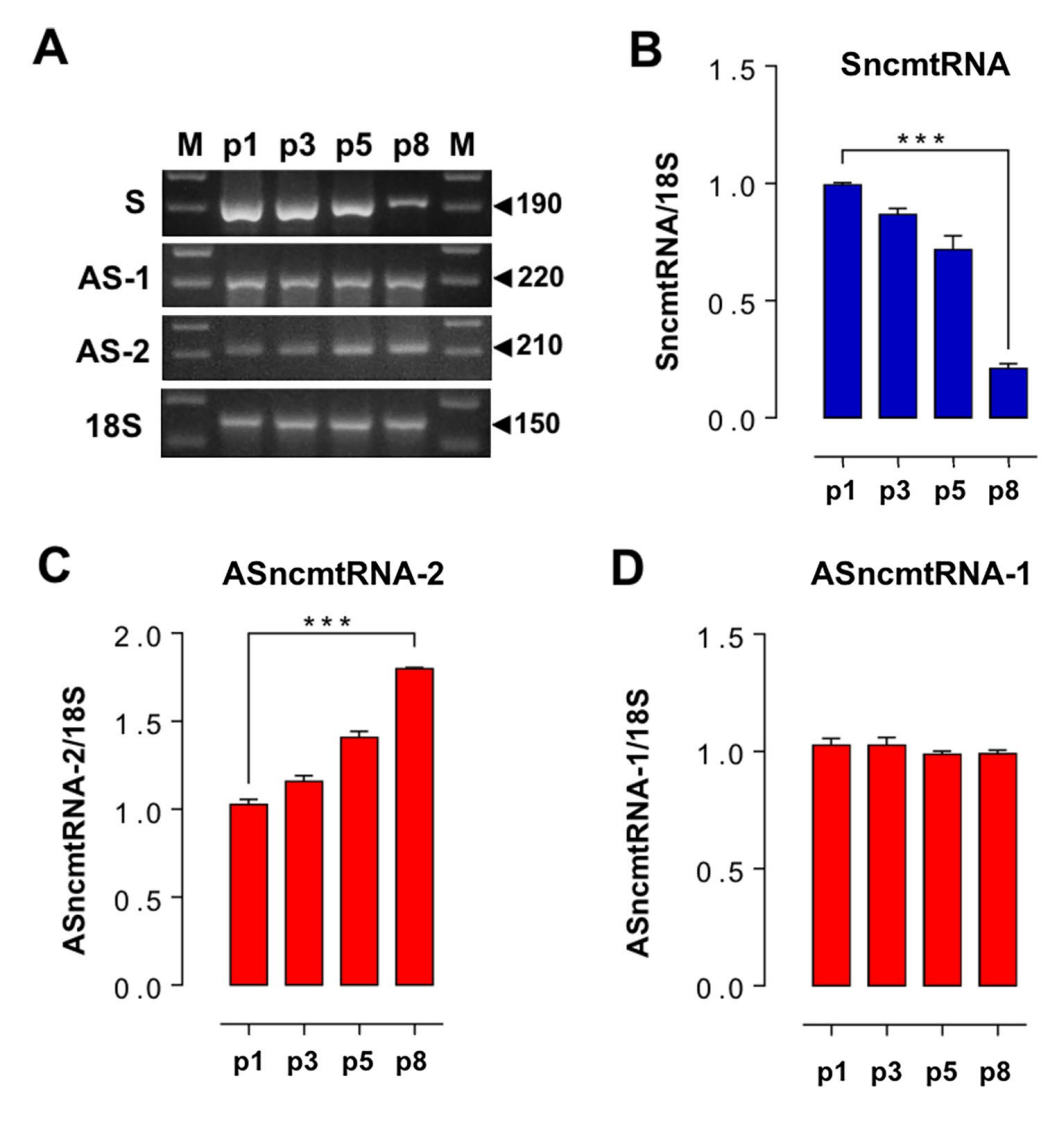
**Changes in ncmtRNA levels in senescent HFK arrested at p8.** The ncmtRNAs were amplified by RT-PCR from total RNA purified from HFK at p1, p3, p5 and p8, using 18S rRNA as an internal control. The experiments were run in triplicate and the relative band density was normalized to 18S for each case. (**A**) Representative gels showing RT-PCR amplification of these transcripts. (**B**) The relative expression of SncmtRNA was downregulated at p8. (**C**) In contrast, ASncmtRNA-2 increased progressively from p1 to senescence at p8 (***p<0.005). (**D**) Relative expression of ASncmtRNA-1 remained constant.

In striking contrast to E2-expressing HFKs (Fig. 2), wild-type and ZsGreen control HFKs exhibit an early replicative arrest. A triplicate analysis of population doublings of HFK and HFK-ZsG showed that these cells arrested at p8 (Fig. 3A, B). A representative cell cycle analysis revealed that HFK and HFK-ZsG at p8 were arrested at G_1_ (Fig. 3C). These cells arrested at G_1_ showed an increase in the activity of the replicative senescence marker SA-β-galactosidase [20] (Fig. 3D, E) together with upregulation of p21 (Fig. 3F, G), as compared to p3 HFK and comparable to HFK cells treated with H_2_O_2_ as positive control of senescence [21] (see Methods). Analogously to p26-arrested HFK-E2 cells, RT-PCR showed that the relative expression of SncmtRNA was strongly downregulated in p8 HFK (Fig. 4A, B), while, conversely, ASncmtRNA-2 was significantly upregulated (Fig. 4A, C) and ASncmtRNA-1 remained constant (Fig. 4A, D).

**Figure 5:**
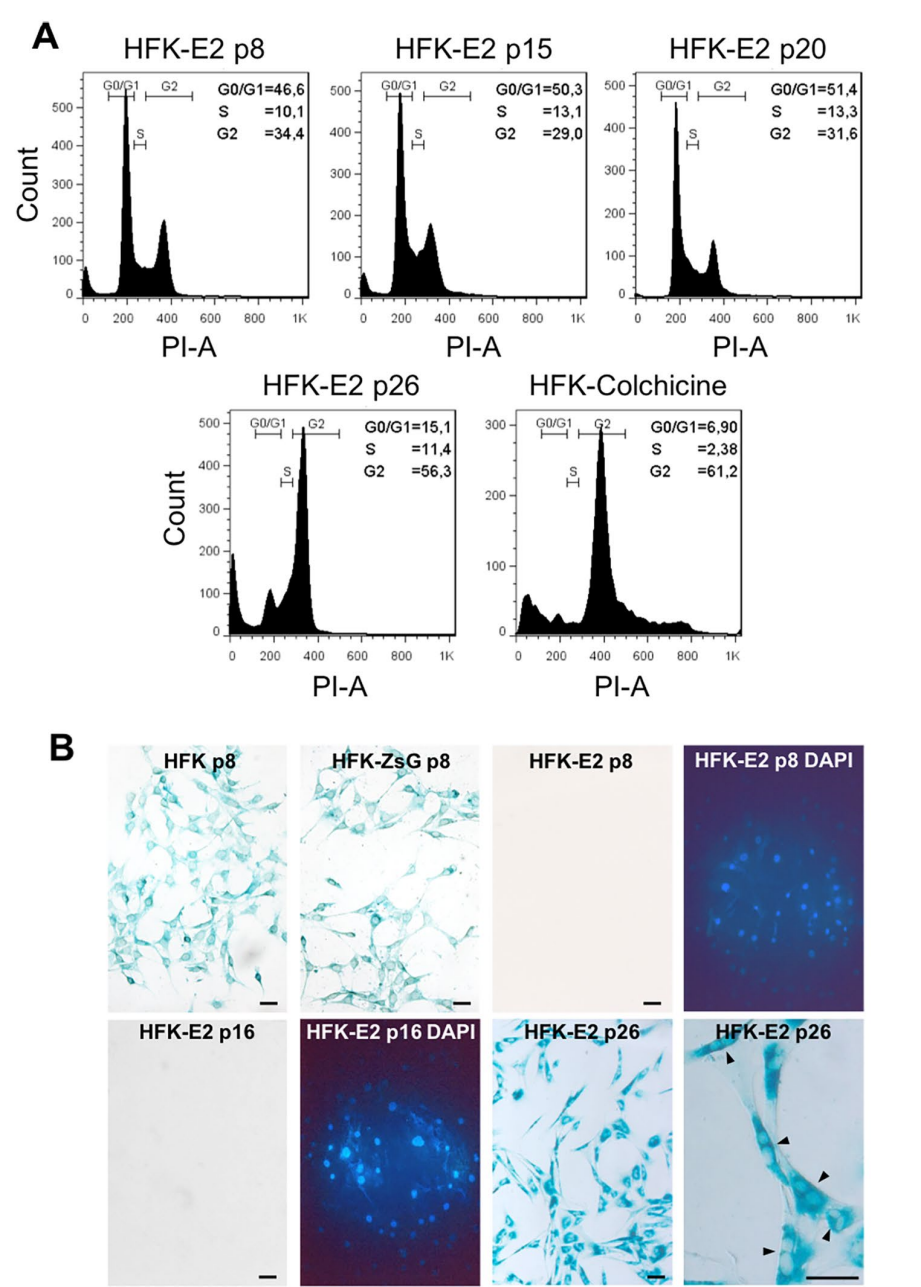
**Spontaneous downregulation of E2 in HPV-18 E2-transduced cells induces replicative senescence.** (**A**) Flow cytometric analysis of HFK-E2 cells at passages 8, 15 and 20 exhibited normal cell cycles with similar proportion of cells at G_0_/G_1_, S and G_2_/M phases. At p26, cells were arrested at G_2_/M, similar to HFK treated with 100 nM colchicine as positive control. (**B**) As shown before, HFK and HFK-ZsG at passage 8 were positive to SA-β-galactosidase activity, whereas HFK-E2 cells at p8 and p16 were negative to this senescence marker (DAPI stain is shown for both fields). At p26, HFK-E2 cells became positive for SA-β-galactosidase staining and a higher magnification shows many binucleated cells (arrowheads). Bars = 50 μm.

In contrast to wild type HFK and HFK-ZsG, cell cycle analysis of HFK-E2 cells showed normal cell cycle distribution of G_1_, S and G_2_/M phases at p8, p15 and p20 with a slight tendency of an increase in the proportion of cells at G_0_/G_1_ (Fig. 5A). However, at p26, cells were arrested at G_2_/M, similar to HFK treated with colchicine, used as positive control (see Methods) (Fig. 5A). At p26, HFK-E2 cells became senescent, as observed by SA-β-galactosidase staining (Fig. 5B). Of note, p8 HFK and HFK-ZsGreen cells were elongated and contained a single nucleus (Fig. 5B, upper left). In contrast, HFK-E2 were negative for SA-β-galactosidase at p8 and p16 but positive at p26 (Fig. 5B). Interestingly, p26 HFK-E2 were binucleated, in agreement with G_2_/M arrest (Fig. 5B, lower right; see arrowheads at higher magnification).

**Figure 6:**
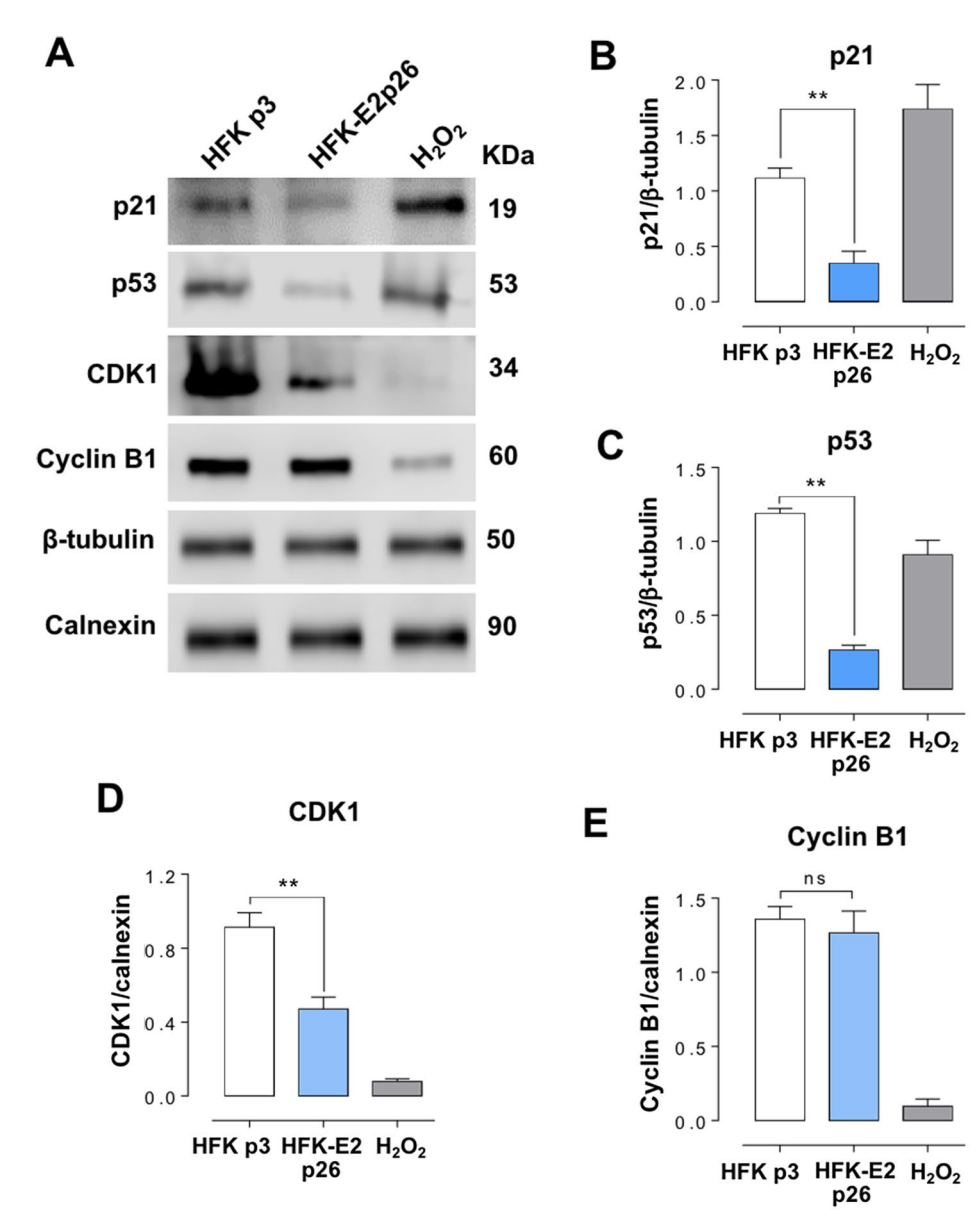
**HFK-E2 cells at p26 inhibit the expression of p21.** (**A**) Western blot of p21, p53, CDK1 and cyclin B1 in HFK at p3 and HFK-E2 at p26, using H_2_O_2_ as positive control of senescence. (**B**) A triplicate Western blot analysis confirms downregulation of p21 in HFK-E2 cells at p26 as compared to p3 HFK (**p<0.01). (**C**) The p53 protein, which is related to p21 expression, is also downregulated (**p<0.01), similar to CDK1 (**D**) (**p<0.01). (**E**) Cyclin B1 expression was not affected in HFK-E2 cells arrested at G_2_/M (ns). Of note, expression of both CDK1 and cyclin B1 is downregulated by treatment with H_2_O_2_. (**p<0.01).

Expression of p21 in HFK-E2 cells at p26 was inhibited as compared to p3 HFK or p3 HFK treated with H_2_O_2_ (Fig. 6A, B). Expression of p21 is dependent on p53 [22–24], and therefore we analyzed whether the expression of this tumor suppressor was also modified. As shown in Fig. 6A and C, the expression of p53 was also downregulated in p26 HFK-E2, compared to p3 HFK. Similarly, the expression of CDK1 (Fig. 6A, D), but not cyclin B1 (Fig. 6A, E), was also downregulated. Notice that treatment of p3 HFK with H_2_O_2_ inhibited the expression of both CDK1 and cyclin B1 (Fig. 6A, D, E).

## DISCUSSION

Here, we demonstrate that expression of HPV-18 E2 protein induces in HFK downregulation of the ASncmtRNAs, establishing a new function for this viral protein during HPV-18-induced cellular transformation. These results confirm our previous suggestion that the E2 protein might be involved in downregulation of the ASncmtRNAs in cells transduced with the complete genome of HPV-18 [14]. However, this model was complicated to reach a definitive conclusion since these cells also express other HPV proteins. Previously, we reported that in different human cancer cell lines and in 17 human cancer biopsies of different origin including uterine cervix carcinoma, the ASncmtRNAs were downregulated and we proposed that these mitochondrial transcripts behave as tumor suppressors [12]. In a challenging review, Bellanger et al. discussed the possibility of pleiotropic characteristic of HPV E2 functioning as either tumor suppressor or oncogene [5]. The present results confirm that the HPV E2 protein behaves as a tumor suppressor at least in cervical cancer and that downregulation of the ASncmtRNAs constitutes an important step in carcinogenesis [12,15–17,25]. However, which cellular factor(s) induces downregulation of the ASncmtRNAs in non-HPV induced cancers remains unclear. Interestingly however, transfection of primary cultured mouse hepatocytes with antigen X of Hepatitis B virus [26] also induces downregulation of the ASncmtRNAs (E. Jeldes et al., unpublished results), suggesting that proteins encoded in other oncogenic viruses may have similar functions.

Previously, we reported that the SncmtRNA and the ASncmtRNAs are synthesized in mitochondria based on the following results: 1) these transcripts exhibit 99% plus identity with the sequence of the human 16S mitochondrial gene. 2) They are detected in isolated mitochondria treated with ribonuclease A to eliminate transcripts attached to the outer membrane. 3) Ethidium bromide and rhodamine 6G, two drugs that interfere with mitochondrial transcription and mtDNA replication, inhibit the expression of these transcripts [11,12]. Taken together, the effect of E2 on the inhibition of the expression of ASncmtRNAs suggests that this viral factor interacts with the organelle. Indeed, it was reported that HPV-18 E2 localizes to the mitochondrial inner membrane, affects cristae morphology and increases mitochondrial ROS production without inducing apoptosis [27]. This report suggests that E2 might affect the expression of the ASncmtRNAs.

Transduction of HFK with HPV-18 E2 extends the replicative lifespan of HFK up to p20-p23 and later, at p26, cells are arrested and become senescent, concomitantly with downregulation of E2 expression. SncmtRNA becomes downregulated upon senescence, both in HFK and HFK-E2 cells, confirming the role of this transcript in cell proliferation, as previously demonstrated with other cell lines [11–15]. On the other hand, ASncmtRNA-2 is gradually upregulated during progression towards senescent arrest, both in wild-type HFK and E2-transduced cells. ASncmtRNA-1, in contrast, does not suffer the same fate; both in HFK and E2-HFK, the expression level of this transcript remains constant, even after reaching replicative senescence. These results suggest that another characteristic of replicative senescence is upregulation of ASncmtRNA-2 and downregulation of the SncmtRNA. Supporting this conclusion is a previous report on human umbilical vein endothelial cells (HUVEC) that become senescent and arrest at G_2_/M after 15 passages. Senescent HUVEC cells express SA-β-galactosidase and p21 and the ASncmtRNA-2 is upregulated [28]. Unfortunately, the expression of SncmtRNA and ASncmtRNA-1 was not evaluated in this report. The differential expression of ASncmtRNA-1 and ASncmtRNA-2 suggests that these two transcripts might play different functions in the cell. Indeed, treatment of the human melanoma cell line SK-MEL-2 with doxorubicin, a drug that induces DNA double-strand breaks [29], upregulates only the expression of ASncmtRNA-1 suggesting that this transcript may be involved in DNA repair (V. Burzio et al. unpublished results).

As described before, senescent HUVEC cells at p15 upregulate the ASncmtRNA-2, together with the expression of hsa-miR-1973 and hsa-miR-4485 [28]. Although in this work we did not determine the presence of these microRNAs, we studied their expression in a different system. We found that knockdown of the ASncmtRNAs in the breast cancer cell line MDA-MB-231 induces a strong increase in the expression of hsa-miR-1973, hsa-miR-4485-3p and hsa-miR-4485-5p, whose sequences are perfectly contained within the inverted repeat of ASncmtRNA-2. An hsa-miR-4485-3p mimic induces a drastic inhibition of cyclin B1 and cyclin D1 (Fitzpatrick et al, submitted).

**Figure 7:**
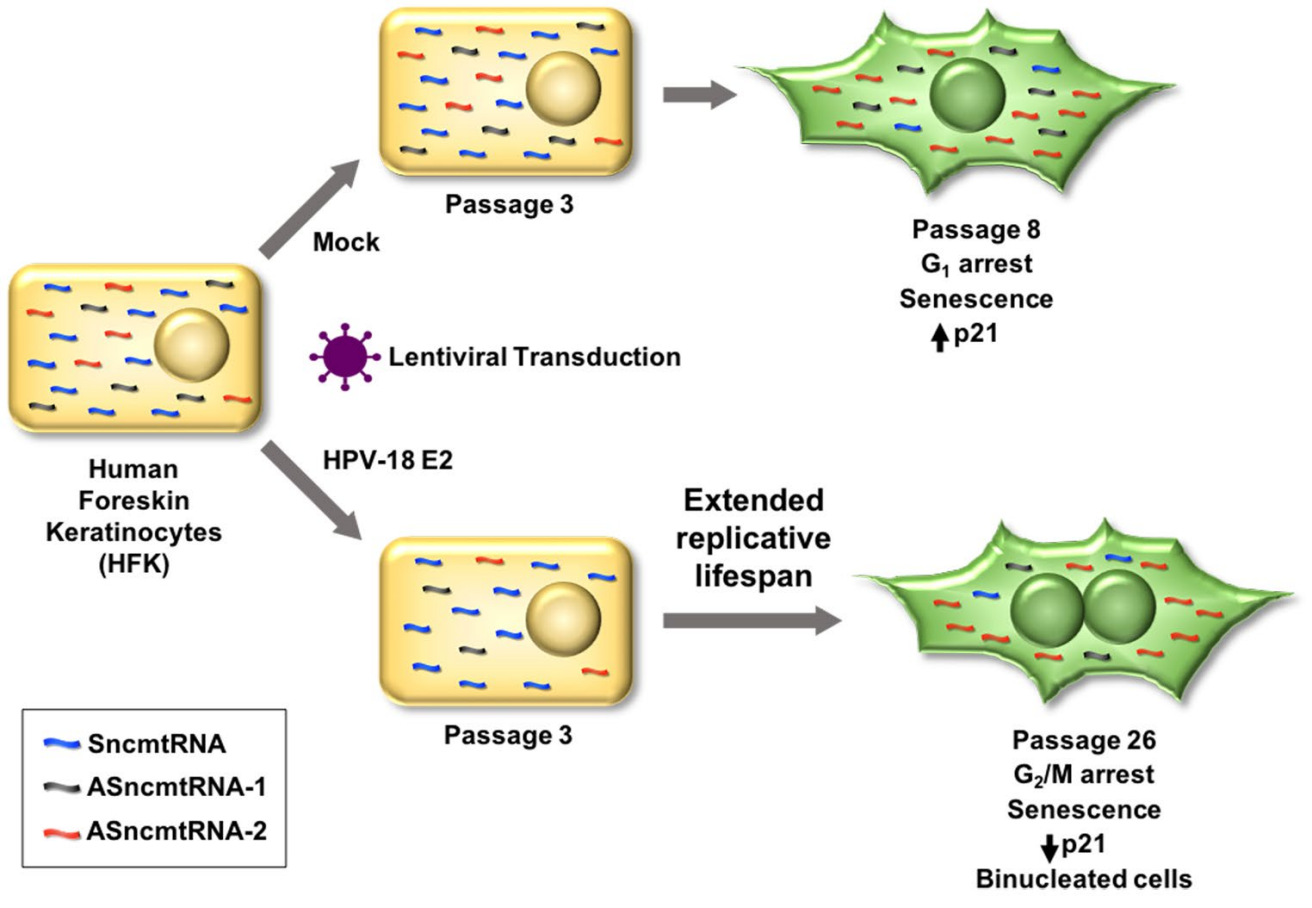
**Lentiviral transduction of HPV-18 E2 induces downregulation of both ASncmtRNA-1 and ASncmtRNA-2 in HFK.** In consequence, replicative lifespan is extended from 8 to 26 passages, when cells become senescent. HFK, at passage 8 (p8), arrest at G_1_, concomitant with increase of the p21 tumor suppressor protein. In contrast, E2-expressing HFK arrest at p26 in G_2_/M, while p21 decreases and cells are binucleated. Remarkably, in both cases, ASncmtRNA-2 is upregulated and SncmtRNA is downregulated upon senescence, while ASncmtRNA-1 remains downregulated in HFK-E2 and constant in HFK and HFK-ZsG.

HFK at p8 and HFK-E2 at p26 display proliferative arrest and exhibit elongated morphology and expression of SA-β-galactosidase, a marker of senescence. However, cell cycle analysis shows a major difference between arrested HFK at p8 and HFK-E2 cells at p26: p8 HFK were arrested at G_1_ while at p26 HFK-E2 cells were arrested at G_2_/M. In addition, arrested HFK cells contain a single nucleus while HFK-E2 cells are binucleated. More important is the notorious difference in the expression of p21, which is upregulated in p8 HFK and downregulated in p26 HFK-E2. Senescence has been described as an irreversible arrest of cells at G_1_ phase [30–33]. However, it is becoming increasingly clear that senescence also takes place at G_2_/M [34–37]. The p21 protein plays an important function as inhibitor of the cyclin-dependent kinases CDK1, CDK2 and CDK4/6 and also as an essential mediator of senescence of cells arrested at G_1_ and G_2_/M [38–42]. The low expression of p21 in HFK-E2 might be explained by downregulation of p53, a tumor suppressor involved in the expression of p21 [39–41]. In addition, we show that CDK1, an essential G_2_/M checkpoint factor of the cell cycle [42,43], is also downregulated, further supporting the G_2_ arrest. Surprisingly, the expression of cyclin B1, activator of CDK1, is not affected by senescence of HFK-E2 cells. A schematic summary of these results is presented in Fig. 7. These observations might be the result of E2-induced cell cycle deregulation and genome instability [44,45]. Interestingly, Hela, HT-3 and CaSki cells infected with a SV40 vector expressing the bovine papillomavirus E2 protein showed downregulation of p53 and p21, besides other proteins involved in cell cycle regulation [46]. However, these results were obtained with previously transformed cells, contrary to the present work where we used normal untransformed keratinocytes as targets for HPV infection.

In conclusion, these results allow us to define cellular phenotypes according to their proliferative status. Previously, we defined three cellular phenotypes according to the expression pattern of the SncmtRNA and the ASncmtRNAs [11–15]. Non-proliferating cells or cells at G_0_ such as human naïve lymphocytes express a trace amount of these transcripts. In contrast, normal proliferating cells express SncmtRNA and the ASncmtRNAs [12]. Remarkably however, tumor cells express the SncmtRNA and downregulate the ASncmtRNAs [12–17]. Here we report that HFK and HFK-E2 reach senescent arrest, together with upregulation of ASncmtRNA-2 and downregulation of the SncmtRNA. Upregulation of the ASncmtRNA-2 was also reported in senescent HUVEC cells arrested at G_2_/M [28]. Therefore, a fourth cellular phenotype is represented by senescent cells, in which the expression of SncmtRNA is downregulated while the ASncmtRNA-2 is upregulated.

## MATERIALS AND METHODS

### Cell culture

Human foreskin keratinocytes (HFK) (Cascade Biologics, Oregon, USA) were cultured in Keratinocyte Serum-free Medium (KSFM; Invitrogen, MA, USA). HPV-18 E2 transduced cells and the HPV-18-transformed 18Nco cell line were cultured in 3:1 media (KSFM + 10%FBS DMEM, Invitrogen, MA, USA). HFK-ZsG cells were grown in KSFM or 3:1 media). All cell lines were cultured at 37ºC and 5% CO_2_ atmosphere until reaching a confluence of 80-90%.

### Construction and production of lentiviral particles encoding HPV-18 E2

HPV-18 E2 ORF was synthetized and cloned by Genscript into the bicistronic lentiviral vector pLVX-IRES-ZsGreen (Clontech, Mountain View, CA, USA). The sequence was partially modified to optimize translation of E2 (Supplementary Fig. 1). Lentiviral particles were produced by co-transfection of lentiviral vector encoding HPV-18 E2, or the empty vector as control (ZsGreen), with the ViraPower packaging system (Invitrogen, MA, USA) into HEK293T cells, using Lipofectamine2000 (Invitrogen, MA, USA) according to manufacturer’s directions [14]. After 48 h, viral particles were harvested and concentrated by ultracentrifugation, and the viral titer was determined by serial dilutions using HEK293FT cells, followed by flow cytometry analysis of ZsGreen expression 72 h post-transduction (BD FacsCanto II).

### Establishment of stable cell lines

HFK were seeded into 6 well plates at a density of 2 x 10^5^ cells/well. At 6 h, cells were transduced with the lentiviral particles encoding HPV-18 E2 and ZsGreen (HFK-E2), or ZsGreen alone (HFK-ZsG), using a multiplicity of infection (MOI) of 5. After 72 h post-transduction, ZsGreen-positive cells were selected by cell sorting (BD FACSAria II). Gating was performed on the brightest cells and collected cells were expanded and used for the following experiments.

### HPV-18 E2 immunofluorescence

Cells (2500/well) were cultured for 24 h in 8 well-chamber slides, then washed in PBS and fixed in 4% p-formaldehyde in PBS for 10 min at room temperature (RT). The slides were washed three times in PBS for 5 min and permeabilized in PBS/0.3% Triton X-100 for 10 min at RT. After 3 washes in PBS for 5 min, cells were blocked in PBS/2% BSA for 30 min. Afterwards, cells were incubated in primary antibody (HPV-18 E2 1:100; Santa Cruz Biotechnology Inc, Paso Robles, CA, USA) in PBS/2% BSA and 0.3% Triton X-100 in a humidified chamber for 1 h at RT. The slides were then washed three times for 5 min in PBS and incubated with Alexa Fluor 568-labeled anti-goat IgG antibody (1:250; Molecular Probes, OR, USA) in 2% BSA for 1 h at RT in the dark. After 2 washes in PBS for 5 min, slides were counterstained with DAPI in PBS for 10 min. Finally, slides were washed in PBS, mounted in Fluorescent Mounting Medium (DAKO) and photographed under an Olympus BX51 epifluorescence microscope.

### RT-PCR

Total RNA was extracted with TRIzol (Invitrogen, MA, USA), according to manufacturer’s instructions. Five µg of RNA were treated with 2 U of TURBO DNA free (Ambion, MA, USA), according to manufacturer’s directions. Reverse transcription was carried out using 100 ng RNA, 50 ng random hexamers, 0.5 mM dNTPs and 200 U reverse transcriptase (M-MLV, Invitrogen, MA, USA) in a final volume of 20 µl. Two µl cDNA was then amplified by PCR in a mix containing 2.5 U GoTaq (Promega, Fitchburg, WI, USA), 1.5 mM MgCl_2_, 0.4 mM dNTPs and 1 µM each primer, in a final volume of 50 µl. PCR amplification was performed as follows: 100°C for 10 min, 70°C for 10 min, 80°C for 10 min and 94°C for 5 min, followed by 26 cycles (ncmtRNAs) or 16 cycles (18S rRNA), consisting of 94°C for 1 min, 58°C for 1 min and 72°C for 1 min. Primers used were 5’AGGTTTAGCCAAACCATT (forw) and 5’AAGGTGGAGTGGGTTTGGGGC’ (rev) for SncmtRNA; 5’ACCGTGCAAAGGTAGCATAATCA (forw) and 5’CAAGAACAGGGTTTGTTAGG (rev) for ASncmtRNA-2; 5’TAGGGATAACAGCGCAATCCTATT (forw) and 5’ CACACCCACCCAAGAACAGGGAGGA (rev) for ASncmtRNA-1; and 5’GTAACCCGTTGAACCCCATT (forw) and 5’CATCCAATCGGTAGTAGCG (rev) for 18S rRNA. For relative quantification, SncmtRNA and ASncmtRNA amplicons were analyzed by densitometry and normalized to 18S rRNA. Experiments were run in triplicate. For HPV-18 E2 full length amplification, 1 µg total RNA, treated as above, was reverse transcribed and 2 µl cDNA were then amplified by PCR in a mix containing 0.5 µl PfuUltra II (Agilent, Santa Clara, CA, USA), 1.5 mM MgCl_2_, 0.4 mM dNTPs and 1 µM each primer in a final volume of 50 µl. PCR amplification was performed as follows: 94°C for 5 min, followed by 30 cycles consisting of 94°C for 1 min, 56°C for 1 min and 72°C for 1,5 min. Primers used were 5’TACGTCTGGGGGTTCTCTGG (forw) and 5’TCACATAGTCATATAGCCGACCAGG (rev).

### Population doubling

Proliferative potential was analyzed by population doubling (PD) analysis. HFK, HFK-ZsG and HFK-E2 cells were cultured at low passage number at 4 x 10^5^ cells per 60-mm plate. When the cells reached 80-90% confluency, they were passaged and re-seeded at the initial density.

### Cell senescence assay

Senescence was determined by staining for SA-β-galactosidase activity with the Senescence Detection Kit (Abcam, Cambridge, MA, USA), according to manufacturer’s directions. Cells (2500/well) were seeded into 8-well chamber slides (Nunc, Invitrogen, MA, USA), cultured for 24 h, washed in PBS and incubated in fixative solution for 15 min at RT. Slides were then washed twice in PBS for 5 min, incubated in staining solution for 24 h at 37°C in a humidified chamber and washed in PBS for 5 min. Afterwards, slides were stained in DAPI in PBS for 10 min, washed in PBS, mounted in Fluorescent Mounting Medium (DAKO, Santa Clara, CA, USA) and analyzed under a fluorescent Olympus BX-51 microscope. SA-β-galactosidase positive cells were counted using ImageJ program. As positive control, cells were treated with 10 µM H_2_O_2_ (Merck, MA, USA) for 48 h [22].

### Western blot

Total cell lysates were subjected to Western blot analysis as described before (9-11). Membranes were probed with antibodies against p21 (mouse monoclonal; 1:400, BD, San Jose, CA, USA), cyclin B1 (mouse monoclonal; 1:350, BD, San Jose, CA, USA), p53 (rabbit polyclonal; 1:300. Cell Signaling, Danvers, MA, USA) or CDK1 (rabbit polyclonal; 1:300 from Cell Signaling Danvers, MA, USA), HPV-18 E2 (rabbit polyclonal Abcam; 1:250, Abcam Cambridge, MA, USA), β-tubulin (rabbit polyclonal Abcam; 1:1000; Cambridge, MA, USA) or calnexin (rabbit polyclonal; 1:500; Novus Biologicals, CO, USA). Primary antibodies were detected using peroxidase-labeled anti-mouse or anti-rabbit IgG (1:5000,). Blots were revealed with the EZ-ECL system (Biological Industries, CT, USA) on a C-DiGit Blot Scanner (LI-COR, Lincoln, NE, USA). The intensity of each band was quantified using ImageJ software (NIH). As positive control for p21 expression, cells were treated with 10 µM H_2_O_2_ for 48 h [22].

### Cell cycle analysis

Stably transduced cells were harvested, fixed in 75% ethanol for 24 h, washed twice in cold PBS and incubated for 30 min at 37ºC in staining solution (3.8 mM sodium citrate, 0.5 μg/ml RNase A (Invitrogen, MA, USA) and 50 μg/ml PI (Invitrogen, MA, USA). PI-stained cells were analyzed on a BD Biosciences FACS Canto Cytometer (Fundación Ciencia & Vida), using the BD FACSDIVA V8.0.1 software for acquisition. HFK treated with 100 nM colchicine (Sigma, St Louis, MO, USA) were used as a G_2_/M arrest control. Cell cycle analysis was performed using FloJo 7.6.1 software.

### Statistics

Student’s *t*-test was used to analyze the significance of each corresponding group of experiments. Significance (*p*-value) was set at the nominal level of p<0.05 or less.

## SUPPLEMENTARY MATERIAL

Supplementary File
