# Supplementary material for: HPV-18 E2 protein downregulates antisense noncoding mitochondrial RNA-2, delaying replicative senescence of human keratinocytes
Source: Aging (Albany NY). 2018 Dec 30;11(1):33–47. doi: 10.18632/aging.101711 (PMC6339806; doi:10.18632/aging.101711)
Supplement: Supplementary File [file aging-11-101711-s001.pdf]

SUPPLEMENTARY MATERIAL

```
ATG CAG ACC CCC AAA GAG ACC CTG TCC GAG AGA CTG TCA TGC GTC CAG GAT
          A   G   G   A       T   G   A CGT TTA AGT       G       C
AAG ATC ATC GAC CAC TAC GAG AAC GAC TCA AAA GAC ATC GAC AGT CAG ATT
  A       A           T   A   T     AGT           A       C   A   A
CAG TAC TGG CAG CTG ATC CGG TGG GAG AAC GCA ATT TTC TTT GCC GCT AGA
      T       A   A   T   T       A   T       A           A   A   G
GAA CAC GGC ATC CAG ACC CTG AAC CAT CAG GTG GTC CCC GCT TAC AAT ATC
      T       A       A TTA       C           G   A   C   T   C   T
TCA AAG AGC AAA GCC CAC AAG GCT ATT GAG CTG CAG ATG GCA CTG CAG GGA
      A   T       A   T   A           A       A       C   T
CTG GCA CAG AGC CGA TAT AAA ACA GAG GAC TGG ACT CTG CAG GAT ACC TGC
  T       A   T       C       C       T       A       A   C   A
GAG GAA CTG TGG AAT ACA GAA CCT ACT CAT TGT TTC AAG AAA GGC GGG CAG
          A                           C   C   T   A       T   C   A
ACC GTG CAG GTC TAC TTT GAC GGG AAC AAG GAT AAT TGC ATG ACA TAT GTG
  A   A   A   A   T       T   C       A   C       T       C       A
GCC TGG GAT TCA GTC TAC TAT ATG ACA GAC GCT GGA ACT TGG GAT AAG ACT
  A       C       G   T           T   T   A       A       C   A   C
GCA ACC TGC GTG AGC CAC AGG GGC CTG TAC TAT GTG AAA GAG GGG TAC AAC
  T       T   A   T           A       T       A   G   A
ACA TTC TAT ATC GAG TTC AAG AGC GAG TGC GAA AAA TAT GGG AAT ACA GGA
  G   T       A   A   T   A   T   A                       C       T
ACT TGG GAG GTG CAC TTC GGC AAC AAT GTC ATT GAC TGC AAC GAT AGC ATG
  G       A   A   T   T   G   T       A       T   T   T   C
TGC TCC ACC TCT GAC GAT ACA GTG TCC GCC ACT CAG CTG GTC AAG CAG CTG
                      C   G   A       T           T   T   A       A
CAG CAT ACA CCC AGT CCT TAC AGC TCC ACC GTG AGC GTC GGA ACC GCC AAA
      C   C           G   T                       G   C       A   G
ACA TAT GGC CAG ACC TCC GCA GCA ACA CGA CCA GGA CAC TGC GGA CTG GCT
  C   C           G   G   T   T           T           T       C   G
GAA AAG CAG CAT TGT GGC CCA GTG AAT CCC CTG CTG GGG GCT GCA ACC CCT
  G                   A   T   C   C   A   T   C   T   A   T   A
ACA GGA AAC AAT AAG CGG AGA CTG CTG TGC TCT GGA AAC ACC ACA CCA ATC
      C       A          A    C   C   T AGT   T       T   G   T   A
ATT CAC CTG AAG GGC GAC CGG AAC AGC CTG AAG TGT CTG AGG TAC CGC CTG
  A   T TTA   A   T     AGA       T TTA   A     TTA C       AGA TT
CGA AAG CAC AGT GAC CAT TAT CGC GAT ATC TCT AGT ACT TGG CAC TGG ACC
      A   T   C       C     AGA       A   A TCC   C       T       A
GGA GCC GGC AAC GAG AAG ACC GGC ATT CTG ACT GTG ACC TAC CAT TCC GAA
  T   A       T   A   A   A   C   A           A   A         AGT
ACT CAG CGG ACC AAA TTT CTG AAC ACT GTG GCT ATC CCT GAC TCC GTG CAG
  A   A AGA   A         TTA   T       T   A   T   A   T AGT   A   A
ATC CTG GTC GGC TAT ATG ACT ATG TGA
  A TTG   G   A   C       A      A
```

Supplementary Figure 1. Nucleotide sequence of HPV-18 E2 used in this study. The sequence was modified to optimize translation of E2. Blue letters correspond to wild type nucleotides. Red letters correspond to optimized nucleotides. 51 nucleotides per line.
